# Association between circulating CD34-positive cells and serum alkaline phosphatase in relation to body mass index for elderly Japanese men

**DOI:** 10.1186/s40101-016-0084-8

**Published:** 2016-01-14

**Authors:** Yuji Shimizu, Shimpei Sato, Jun Koyamatsu, Hirotomo Yamanashi, Mako Nagayoshi, Koichiro Kadota, Kazuto Tsuruda, Naomi Hayashida, Norio Abiru, Hironori Yamasaki, Noboru Takamura, Takahiro Maeda

**Affiliations:** 1 Department of Community Medicine, Nagasaki University Graduate School of Biomedical Sciences, Nagasaki, Japan; 2 Department of Island and Community Medicine , Nagasaki University Graduate School of Biomedical Sciences, Nagasaki, Japan; 3 Department of Laboratory Medicine, Nagasaki University Hospital, Nagasaki, Japan; 4 Division of Strategic Collaborative Research Center for Promotion of Collaborative Research on Radiation and Environment Health Effects, Nagasaki University Graduate School of Biomedical Sciences, Nagasaki, Japan; 5 Department of Endocrinology and Metabolism, Unit of Translational Medicine, Nagasaki University Graduate School of Biomedical Sciences, Nagasaki, Japan; 6 Center for Health and Community Medicine, Nagasaki University, Nagasaki, Japan; 7 Department of Global Health, Medicine and welfare, Atomic bomb Disease Institute, Nagasaki University Graduate School of Biomedical Sciences, Nagasaki, Japan

## Abstract

**Background:**

Recent studies have confirmed an association between bone metabolism and vascular homeostasis. However, no study has examined the relationship between serum alkaline phosphatase (ALP) (a marker of bone metabolism) and circulating immature cell such as CD34-positive cells (a marker of vascular homeostasis).

**Methods:**

We conducted a cross-sectional study of this association in 272 elderly Japanese men (60–79 years). Because low body mass index (BMI) status is a known characteristic of Japanese with a high incidence rate of stroke, we used a stratified analysis based on BMI.

**Results:**

Multivariable linear regression analysis adjusted for confounding factors showed a significant correlation between serum ALP and the number of circulating CD34-positive cells, especially for participants with low BMI (<23 kg/m^2^). The parameter estimates (*β*) and 95 % confidence intervals (CI) for one standard deviation increments in serum ALP levels (62 IU/L) for the circulating CD34-positive cell count were *β* = 0.25 (0.04, 0.45) for total subjects, *β* = 0.45 (0.16, 0.75) for participants with low BMI (<23 kg/m^2^), and *β* = 0.04 (−0.25, 0.34) for participants with high BMI (≥23 kg/m^2^).

**Conclusion:**

Serum ALP correlates positively with circulating CD34-positive cells among a general population of elderly Japanese men, especially those with low BMI (<23 kg/m^2^). These findings suggest that serum ALP levels may constitute an efficient tool for estimating the risk of insufficient vascular homeostasis, especially for participants with relatively few classical cardiovascular risk factors.

## Introduction

A previous study reported identifying a positive association between elevated serum alkaline phosphatase (ALP) and atherosclerosis [1]. Other prospective studies have identified an association between high serum ALP levels and mortality from all causes or cardiovascular disease for subjects with previous myocardial infarction [2] and previous stroke [3], in a clinic population [4], as well as a general population [2]. However, in a retrospective study of ours as part of the Circulatory Risk in Communities Study (CIRCS), we reported that not only high but also low serum ALP levels may be a predictor of risk of stroke for Japanese [5]. Other recent studies reported detecting associations between bone metabolism and vascular homeostasis [6–13] in view of the fact that hematopoietic stem cells (immature cell such as CD34-positive cell) derived from the bone marrow play a major role in vascular homeostasis [7–9]. Because osteoblasts, whose activity can be evaluated in terms of bone-type ALP expression [14, 15], regulate the production of hematopoietic stem cells in the bone marrow [10–12], serum ALP levels may be related to vascular homeostatic activity. Another study reported that a reduction in the number of endothelial progenitor cells (CD34-positive cells) is associated with an increase in the number of infarctions in patients with ischemic stroke [16].

However, no study has examined whether the number of circulating CD34-positive cells is associated with serum ALP levels. In this connection, the prevalence of obesity in adults defined by a body mass index (BMI) of ≥30 kg/m^2^ was only 3.6 % in Japan whereas it was 35.3 % in the USA in 2012 [17]. Nevertheless, the incidence of stroke and stroke mortality is higher in East Asian countries than in Western countries [18]. Since only a study of a Japanese general population has identified a risk of stroke in association with lower serum ALP level [5], the association between serum ALP level and the number of circulating CD34-positive cells may be strongly influenced by BMI status.

To investigate the validity of this hypothesis, we conducted a cross-sectional study of elderly Japanese men aged 60–79 years.

## Materials and methods

### Subjects

Written consent forms were available in Japanese to ensure comprehensive understanding of the study objectives, and informed consent was signed by all participants. Since postmenopausal changes such as osteopenia, osteoporosis [19], and dyslipidemia are known to have a major impact on serum ALP levels, women were not considered suitable subjects for an analysis of the association between serum ALP and circulating CD34-positive cells. The survey population comprised 409 60–79-year-old men who underwent a general medical check-up as recommended by the Japanese government. They were residents of rural communities in the Goto Islands in western Japan and participated in this study in 2013 and 2014.

Persons with missing data (*n* = 132) were excluded. To avoid the effect of bone disorders such as osteoporosis, bone fracture, and bone tumor, we also excluded persons with a high serum ALP level (>400 IU/L) (*n* = 5). The remaining 272 men with a mean age of 66.6 years (standard deviation (SD) ±4.4; range 60–79), none of whom were taking medication for anemia, were enrolled in this study, which was approved by the Ethics Committee for Human Use of Nagasaki University (project registration number 0501120073).

### Data collection and laboratory measurements

Body weight and height were measured with an automatic body composition analyzer (BF-220; Tanita, Tokyo, Japan) when blood samples were obtained, and BMI (kg/m^2^) was calculated. Systolic blood pressure and diastolic blood pressure were recorded at rest.

Fasting blood samples were collected in a heparin sodium tube and a siliconized tube. Fresh samples (within 24 h from obtained) from the former were used for determining the number of CD34-positive cells. BD (Beckton Dickinson Biosciences) Trucount™ technology for an accurate and reproducible single platform assay with International Society of Hematotherapy and Graft Engineering (ISHAGE) guidelines [20, 21] which was supported by an automated software on the BD FACSCant™ II system was used to measuring circulating CD34-positive cells. Serum samples were separated for the measurement of concentrations of alkaline phosphatase (ALP), aspartate aminotransferase (AST), serum alanine aminotransferase (ALT), and γ-glutamyltranspeptidase (γ-GTP) with the Japanese Society of Clinical Chemistry (JSCC) standardization method.

Triglyceride (TG) and creatinine were measured with the enzyme method. HDL-cholesterol (HDL), calcium (Ca), and phosphorus (P) were measured by direct method, Arsenazo III method, and molybdate direct method, respectively. Hemoglobin A1c (HbA_1c_) was measured with the latex coagulation method. The glomerular filtration rate (GFR) was estimated by means of an established method with three variations recently proposed by a working group of the Japanese Chronic Kidney Disease initiative [22]. According to this adapted version, GFR (mL/min/1.73 m^2^) = 194 × (serum creatinine (enzyme method))^−1.094^ × (age)^−0.287^.

### Statistical analysis

Characteristics of the study participants were expressed in terms of BMI status.

Simple correlation analyses of serum ALP and other variables affecting BMI status, as well as of the numbers of CD34-positive cells and other variables, were performed. Multiple linear regression analysis was performed to determine the correlations between the number of circulating CD34-positive cells and one SD increment in ALP (62 IU/L). Because ALP is expressed differently in various tissues, with notably high concentrations in the liver, bone, and kidneys [14], we designed the analyses to allow for adjustments for classical cardiovascular risks factors and factors associated with the liver, as well as factors that might be directly involved in bone metabolism. The latter factors comprised age, systolic blood pressure (mmHg), diastolic blood pressure (mmHg), body mass index, smoking status (never smoker, former smoker, current smoker), alcohol consumption (never drinker, former drinker, current drinker [<23, 23–45, 46–68, ≥69 g/week]), HbA1c (%), HDL (mg/dL), TG (mg/dL), AST (IU/L), ALT (IU/L), γ-GTP (IU/L), Ca (mg/dL), P (mg/dL), and GFR (mL/min/1.72 m^2^).

All statistical analyses were performed with the SAS system for Windows (version 9.3; SAS Inc., Cary, NC). Probability values less than 0.05 were considered indicative of statistical significance.

## Results

Table 1 shows the characteristics of the study population. Compared to participants with low BMI (defined as <23 kg/m^2^), participants with high BMI (defined as ≥23 kg/m^2^) show significantly higher systolic blood pressure, diastolic blood pressure, TG, ALT, γ-GTP, HbA1c, and serum creatinine while significantly lower HDL and GFR.

**Table 1:** Age-adjusted characteristics of study population by BMI status

	BMI <23 kg/m^2^	BMI ≥23 kg/m^2^	*p* value
No. of participants	122	150	
Age, years	66.5 ± 4.4	66.7 ± 4.4	
Circulating CD34-positive cells, cells/μL	1.33	1.55	0.231
Serum alkaline phosphatase (ALP), UI/L	239	225	0.065
Systolic blood pressure, mmHg	134	139	0.016
Diastolic blood pressure, mmHg	81	87	<0.001
Body mass index (BMI), kg/m^2^	20.9	25.7	<0.001
Current drinker, %	76.4	84.5	0.088
Current smoker, %	28.4	20.3	0.111
Serum HDL-cholesterol (HDL), mg/dL	61	54	<0.001
Serum triglycerides (TG), mg/dL	104	127	0.046
Hemoglobin A1c (HbA1c), %	5.6	5.8	0.027
Serum aspartate aminotransferase (AST), IU/L	24	26	0.105
Serum alanine aminotransferase (ALT), IU/L	20	26	<0.001
Serum γ-glutamyltranspeptidase (γ-GTP), IU/L	36	53	0.007
Serum calcium (Ca), mg/dL	9.3	9.3	0.208
Serum phosphorus (P), mg/dL	3.3	3.3	0.455
Serum creatinine, mg/dL	0.82	0.86	0.016
Glomerular filtration rate (GFR), mL/min/1.73 m^2^	75.1	71.2	0.023

Age: mean ± standard deviation

Simple correlation analysis was used to determine associations of serum ALP with other variables affecting BMI status (Table 2). For total subjects, serum ALP correlated positively with age, TG, and AST and negatively with HDL and alcohol consumption. Stratification of those correlations by BMI status disclosed that ALP correlated with TG for both participants with low BMI and high BMI. We also found that ALP correlated positively with AST and γ-GTP and negatively with alcohol consumption and HDL for high BMI and positively with age for low BMI.

**Table 2:** Simple correlation analysis of ALP and other variables

	Total subjects		BMI <23 kg/m^2^		BMI ≥23 kg/m^2^	
	*r*	*p*	*r*	*p*	*r*	*p*
No. of participants	272		122		150	
Age	0.16	0.010	0.22	0.017	0.11	0.165
Systolic blood pressure	0.11	0.072	0.10	0.287	0.16	0.056
Diastolic blood pressure	0.06	0.364	0.08	0.383	0.09	0.255
Body mass index (BMI)	−0.08	0.190	−0.14	0.115	0.12	0.156
Alcohol consumption	−0.20	<0.001	−0.13	0.149	−0.24	0.003
Smoking status	0.05	0.423	0.05	0.567	0.03	0.698
Serum HDL-cholesterol (HDL)	−0.19	0.002	−0.13	0.140	−0.30	<0.001
Serum triglycerides (TG)	0.29	<0.001	0.24	0.008	0.36	<0.001
Hemoglobin A1c (HbA1c)	0.04	0.536	0.04	0.635	0.06	0.454
Serum aspartate aminotransferase (AST)	0.13	0.033	0.05	0.583	0.20	0.013
Serum alanine aminotransferase (ALT)	0.08	0.205	0.04	0.670	0.15	0.061
Serum γ-glutamyltranspeptidase (γ-GTP)	0.09	0.119	0.01	0.879	0.16	0.046
Serum creatinine	−0.07	0.279	−0.12	0.190	0.002	0.980
Glomerular filtration rate (GFR)	0.07	0.263	0.09	0.306	−0.02	0.773
Serum calcium (Ca)	−0.11	0.078	−0.12	0.175	−0.08	0.325
Serum phosphorus (P)	−0.08	0.191	−0.10	0.266	−0.07	0.385

Alcohol consumption [never drinker, former drinker, current drinker (<23, 23–45, 46–68, ≥69 g/week)] and smoking status (never smoker, former smoker, current smoker)

**Fig. 1:**
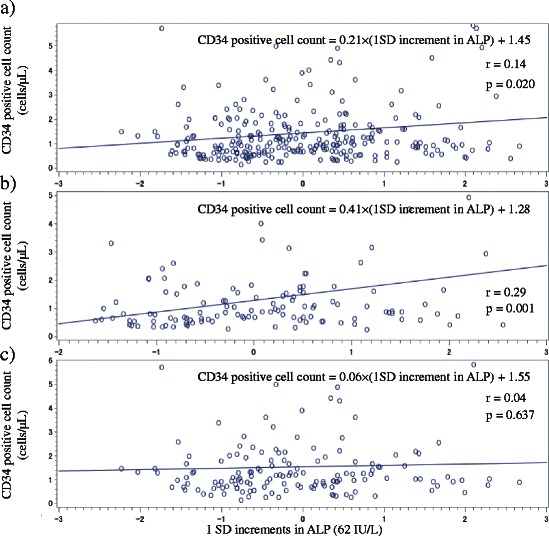
Relationship between circulating CD34-positive cells and one SD increment in serum ALP in **a** total subjects, **b** those with low BMI (<23 kg/m^2^), and **c** those with high BMI (≥23 kg/m^2^)

Simple correlation analyses of the number of circulating CD34-positive cells and other variables are shown in Table 3. The cells showed a significantly positive correlation with serum ALP for total (*r* = 0.14, *p* = 0.020) and low BMI (*r* = 0.29, *p* = 0.001) but not for high BMI (*r* = 0.04, *p* = 0.637). We also determined the correlation between number of circulating CD34-positive cells and a one SD increment in serum ALP (Fig. 1).

**Table 3:** Simple correlation analysis of number of circulating CD34-positive cells and other variables

	Total subjects		BMI <23 kg/m^2^		BMI ≥23 kg/m^2^	
	*r*	*p*	*r*	*p*	*r*	*p*
No. of participants	272		122		150	
Age	−0.04	0.460	0.09	0.349	−0.15	0.072
Systolic blood pressure	−0.04	0.503	−0.02	0.856	−0.08	0.309
Diastolic blood pressure	−0.01	0.894	0.06	0.520	−0.09	0.250
Body mass index (BMI)	0.03	0.642	0.04	0.666	−0.10	0.202
Alcohol consumption	−0.03	0.623	−0.04	0.652	−0.04	0.646
Smoking status	−0.03	0.611	−0.002	0.983	−0.05	0.576
Serum HDL-cholesterol (HDL)	−0.03	0.633	0.04	0.689	−0.06	0.466
Serum triglycerides (TG)	0.10	0.073	0.08	0.353	0.11	0.171
Hemoglobin A1c (HbA1c)	0.10	0.118	−0.02	0.829	0.18	0.028
Serum aspartate aminotransferase (AST)	−0.05	0.490	−0.07	0.459	−0.04	0.631
Serum alanine aminotransferase (ALT)	0.05	0.419	−0.05	0.579	0.07	0.364
Serum γ-glutamyltranspeptidase (γ-GTP)	0.01	0.863	0.04	0.635	−0.02	0.837
Glomerular filtration rate (GFR)	−0.03	0.616	−0.04	0.696	−0.01	0.902
Serum calcium (Ca)	0.002	0.978	−0.01	0.932	−0.001	0.989
Serum phosphorus (P)	−0.04	0.563	−0.10	0.240	0.02	0.790
Serum alkaline phosphatase (ALP)	0.14	0.020	0.29	0.001	0.04	0.637

Alcohol consumption [never drinker, former drinker, current drinker (<23, 23–45, 46–68, ≥69 g/week)] and smoking status (never smoker, former smoker, current smoker)

As shown in Table 4, multiple linear regression analysis adjustment for confounding factors showed that the number of circulating CD34-positive cells correlated positively with a one SD increment in serum ALP for total (*β* = 0.25, *p* = 0.019) and low BMI (*β* = 0.45, *p* = 0.003) but not for high BMI (*β* = 0.04, *p* = 0.772).

**Table 4:** Multiple linear regression analysis of circulating CD34-positive cells with relevant factors adjusted for confounding factors

	Total subjects			BMI <23 kg/m^2^			BMI ≥23 kg/m^2^		
	*β*	95 % CI	*p*	*β*	95 % CI	*p*	*β*	95 % CI	*p*
No. of participants	272			122			150		
Age	−0.02	(−0.06, 0.03)	0.467	0.02	(−0.04, 0.09)	0.485	−0.07	(−014, −0.004)	0.038
Systolic blood pressure	−0.01	(−0.02, 0.01)	0.462	−0.02	(−0.04, 0.01)	0.175	0.005	(−0.02, 0.03)	0.671
Diastolic blood pressure	−0.002	(−0.03, 0.02)	0.852	0.02	(−0.02, 0.06)	0.360	−0.02	(−0.06, 0.01)	0.152
Body mass index (BMI)	0.01	(−0.06, 0.09)	0.749	0.10	(−0.09, 0.29)	0.291	−0.11	(−0.25, 0.03)	0.134
Alcohol consumption	0.02	(−0.09, 0.13)	0.682	−0.02	(−0.19, 0.14)	0.770	−0.01	(−0.16, 0.14)	0.906
Smoking status	−0.13	(−0.42, 0.16)	0.390	0.04	(−0.41, 0.48)	0.874	−0.31	(−0.72, 0.09)	0.129
Serum HDL-cholesterol (HDL)	0.01	(−0.01, 0.02)	0.400	0.02	(−0.005, 0.04)	0.138	−0.003	(−0.03, 0.02)	0.800
Serum triglycerides (TG)	0.001	(−0.001, 0.003)	0.333	0.001	(−0.003, 0.005)	0.736	0.002	(−0.001, 0.01)	0.178
Hemoglobin A1c (HbA1c)	0.14	(−0.16, 0.44)	0.367	−0.09	(−0.55, 0.37)	0.704	0.44	(−0.01, 0.89)	0.056
Serum aspartate aminotransferase (AST)	−0.03	(−0.06, 0.01)	0.104	−0.03	(−0.09, 0.02)	0.241	−0.03	(−0.07, 0.02)	0.252
Serum alanine aminotransferase (ALT)	0.02	(−0.01, 0.04)	0.250	0.01	(−0.04, 0.06)	0.767	0.01	(−0.02, 0.05)	0.387
Serum γ-glutamyltranspeptidase (γ-GTP)	0.001	(−0.004, 0.005)	0.821	0.004	(−0.01, 0.02)	0.468	0.001	(−0.004, 0.01)	0.701
Glomerular filtration rate (GFR)	−0.004	(−0.02, 0.01)	0.571	−0.004	(−0.02, 0.02)	0.703	−0.003	(−0.02, 0.02)	0.775
Serum calcium (Ca)	0.01	(−0.56, 0.58)	0.970	0.06	(−0.82, 0.94)	0.889	−0.11	(−0.88, 0.66)	0.776
Serum phosphorus (P)	−0.13	(−0.56, 0.31)	0.565	−0.06	(−0.76, 0.63)	0.854	0.05	(−0.56, 0.65)	0.880
One SD increment in serum alkaline phosphatase (ALP)	0.25	(0.04, 0.45)	0.019	0.45	(0.16, 0.75)	0.003	0.04	(−0.25, 0.34)	0.772

Alcohol consumption [never drinker, former drinker, current drinker (<23, 23–45, 46–68, ≥69 g/week)] and smoking status (never smoker, former smoker, current smoker)

## Discussion

The major finding of this study of elderly Japanese men was that serum ALP significantly and positively correlates with circulating CD34-positive cells, especially for participants with low BMI (<23 kg/m^2^). This suggests that serum ALP levels may constitute an efficient tool for estimating the risk of insufficient vascular homeostasis, especially for participants with relatively few classical cardiovascular risk factors.

The mechanism most likely to be responsible for the association between serum ALP and circulating CD34-positive cell is impaired vascular homeostasis since hematopoietic stem cells derived from the bone marrow play a major role in vascular homeostasis [7–9]. Moreover, because osteoblasts (whose activity can be assessed in terms of bone-type ALP expression [14, 15]) regulate the production of hematopoietic stem cells in the bone marrow [10–12], serum ALP level may correlate with vascular homeostatic activity, and a prospective study reported a strong association between a reduced number of endothelial progenitor cells (CD34-positive and KDR-positive cell) and risk of cardiovascular disease [23]. Yet another study found an association of a reduction in the number of endothelial progenitor cells (CD34-positive cells) with an increase in the number of infarctions >5 mm in diameter, but no such association with atherosclerosis in carotid arteries of patients with ischemic stroke [16]. Endothelial progenitor cells contribute to vascular repair [24, 25], and a reduction in the number of these cells may thus increase the risk of stroke. Results of another retrospective CIRCS study of ours indicated that not only higher but also lower serum ALP levels may be a predictor of the risk of stroke among Japanese [5]. These findings seem to be, at least in part, compatible with those of our present study.

Since hematopoietic stem cells have also been reported to participate in the pathogenesis of atherosclerosis [13], an increase in serum ALP may be associated with the deterioration of arterial stiffness. A previous study reported that, independent of other traditional cardiovascular risk factors, elevated serum ALP is associated with atherosclerosis evaluated by means of ankle-brachial blood pressure index [1]. However, in our subsequent analysis (*n* = 191), we could not find any association between serum ALP and arterial stiffness evaluated by cardio-ankle vascular index (CAVI) (*r* = −0.02; *P* = 0.874). Since our study population consists of elderly subjects and side population hematopoietic bone marrow stem cells diminish as individuals age [26, 27], so that even vascular injury can activate bone marrow metabolism, the bone marrow could not be activated as it would be in younger participants while the atherosclerotic lesion might have originated in their younger years. Furthermore, extensive reduction in bone marrow activity also may have a confounding effect on the aforementioned associations. The significant correlation between serum ALP and circulating CD34-positive cells observed in our study was therefore restricted to low BMI. In addition, participants with high BMI possessed many more classical cardiovascular risk factors such as metabolic syndrome than did those with lower BMI (<23 kg/m^2^). In this connection, the World Health Organization (WHO) has identified BMI ≥23 kg/m^2^, which corresponds to the median BMI in our study, as an indicator for enhanced risk of disease for Asian populations [28]. Another possible mechanism that might explain why a significant correlation between serum ALP and the number of circulating CD34-positive cells is limited to low BMI (<23 kg/m^2^) is the fact that the factors that determine the serum ALP levels are different. Serum ALP is classified in terms of isoenzymes, that is, liver-type, bone-type, and intestinal-type [29]. For the subjects with high BMI in our study, serum ALP levels are much more likely to be influenced by factors associated with liver condition such as alcohol consumption, HDL, AST, and γ-GTP than for the subjects with low BMI. Furthermore, the simple correlation analyses showed significant correlations between the number of circulating CD34-positive cells and HbA1c for high BMI but not for low BMI (≥23 kg/m^2^). Since HbA1c levels were significantly higher for subjects with high BMI than for those with low BMI, HbA1c could be an important stimulating factor for circulating CD34-positive cell production in subjects with high BMI but not with low BMI.

Our findings should be interpreted with some caution. First, because the ALP isoenzyme was not measured [29], we could not assess which type of ALP correlated with circulating CD34-positive cells. However, in the present study, we detected a significant correlation between serum ALP and the circulating number of CD34-positive cells. Second, because of the limited number of participants, we could not perform a drinking status-specific analysis even though serum ALP is known to be associated with drinking status [5, 30]. However, a significant correlation between serum ALP and circulating CD34-positive cells was observed even after adjustment for alcohol consumption. Furthermore, the significant correlation between serum ALP and alcohol consumption was observed only among participants with high BMI.

Although the correlation between serum ALP levels and circulating CD34-positive cells was shown to be independent of the traditional risk factors, we did not adjust for other potential confounding factors whose values were associated with serum ALP, such as caloric, protein, vitamin C, magnesium, and zinc deficiencies [31].

In conclusion, we showed that serum ALP correlates positively with circulating CD34-positive cells among a general population of elderly Japanese men, especially those with low BMI (<23 kg/m^2^). These findings suggest that serum ALP levels may constitute an efficient tool for estimating the risk of insufficient vascular homeostasis, especially for participants with comparatively few classical cardiovascular risk factors.

## Abbreviations

ALP: alkaline phosphatase
ALT: serum alanine aminotransferase
AST: aspartate aminotransferase
BMI: body mass index
Ca: calcium
CAVI: cardio-ankle vascular index
CI: confidence intervals
CIRCS: Circulatory Risk in Communities Study
GFR: glomerular filtration rate
HbA1c: hemoglobin A1c
HDL: HDL-cholesterol
JSCC: Japanese Society of Clinical Chemistry
P: phosphorus
TG: triglyceride
WHO: World Health Organization
*β*: parameter estimates
γ-GTP: γ-glutamyltranspeptidase
